# *In situ* synthesis of monolayer graphene on silicon for near-infrared photodetectors

**DOI:** 10.1039/c9ra06792b

**Published:** 2019-11-18

**Authors:** Pengcheng Xiang, Gang Wang, Siwei Yang, Zhiduo Liu, Li Zheng, Jiurong Li, Anli Xu, Menghan Zhao, Wei Zhu, Qinglei Guo, Da Chen

**Affiliations:** Department of Microelectronic Science and Engineering, School of Physical Science and Technology, Ningbo University Ningbo 315211 P. R. China gangwang@nbu.edu.cn chenda@nbu.edu.cn; State Key Laboratory of Functional Materials for Informatics, Shanghai Institute of Microsystem and Information Technology Shanghai 200500 P. R. China; State Key Laboratory of Integrated Optoelectronics, Institute of Semiconductors, Chinese Academy of Sciences Beijing 100083 P. R. China; Center of Nanoelectronics and School of Microelectronics, Shandong University Jinan 250100 P. R. China qlguo@sdu.edu.cn; College of Materials Science and Opto-Electronic Technology, University of Chinese Academy of Sciences Beijing 100049 P. R. China; Department of Materials Science, Fudan University Shanghai 200433 P. R. China

## Abstract

Direct integration of monolayer graphene on a silicon (Si) substrate is realized by a simple thermal annealing process, involving a top copper (Cu) layer as the catalyst and an inserted polymethylmethacrylate (PMMA) as the carbon source. After spin-coating the PMMA carbon source on the Si substrate, the Cu catalyst was deposited on PMMA/Si by electron beam evaporation. After that, graphene was directly synthesized on Si by decomposition and dehydrogenation of PMMA and the catalyzation effect of Cu under a simple thermal annealing process. Furthermore, under an optimized growth condition, monolayer graphene directly formed on the Si substrate was demonstrated. Utilizing the as-grown graphene/Si heterojunction, near-infrared photodetectors with high detectivity (∼1.1 × 10^10^ cm Hz^1/2^ W^−1^) and high responsivity (50 mA W^−1^) at 1550 nm were directly fabricated without any post-transfer process. The proposed approach for directly growing graphene on silicon is highly scalable and compatible with present nano/micro-fabrication systems, thus promoting the application of graphene in microelectronic fields.

## Abstract 2

Direct integration of monolayer graphene on a silicon (Si) substrate is realized by a simple thermal annealing process, involving a top copper (Cu) layer as the catalyst and an inserted polymethylmethacrylate (PMMA) as the carbon source.

## Graphical abstract

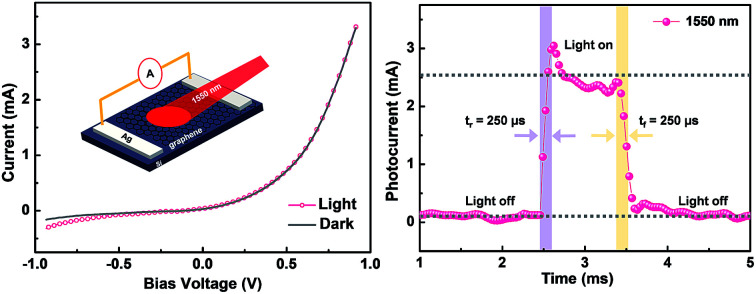

## 1. Introduction

Owing to its excellent mechanical and electrical properties, graphene has been a focus of research since it was first demonstrated by mechanical exfoliation from graphite in 2004.^1,2^ After that, researchers have presented various feasible strategies to produce graphene films, as one of which, chemical vapor deposition (CVD), has been extensively adopted to grow large-area and high-crystallinity graphene on metal substrates.^3^ Nevertheless, graphene films deposited on metal surfaces must be transferred onto other substrates, like Si, Ge or SiO_2_, for subsequent device fabrication, where defects are always formed in the graphene films that degrade their qualities and properties.^4–6^ Therefore, the capability of synthesizing graphene on any desired substrate without post-transfer processing should be urgently demanded.^7^ Motivated by the above, graphene directly grown on non-metal materials such as germanium, SiO_2_, h-BN, and Al_2_O_3_, has been recently demonstrated.^8–12^ However, single-crystalline Si, the most common commercial semiconductor, attracted scant attention when utilized as the substrate for direct synthesis of graphene. Previous works showed that the pre-formation of Si carbide during the PMMA decomposition process weakens the nucleation and growth of graphene.^13,14^ Although solid source molecular-beam epitaxy or CVD without a metallic catalyst may also provide possibilities, only graphitic films have been accomplished.^15–17^

In this work, we report a practical and straightforward technology to directly synthesize graphene on Si substrates. The key idea of this approach is to form a uniform PMMA on Si, followed by depositing a Cu layer on the PMMA as the catalyst. Notably, the PMMA appears to have a dual-role: (i) as the carbon source to synthesize graphene film; (ii) as the barrier to prevent Cu diffusing into Si substrate. We should stress that Cu atom residuals are still found in silicon after graphene synthesis although the fraction is meager. According to the optimized growth condition, the highest annealing temperature should not exceed 900 °C, aiming to prevent the wetting and evaporation of Cu layer and the annealing time is shortened to be 13 min. Finally, high-performance near-infrared photodetectors are directly fabricated by utilizing the as-grown graphene/Si heterojunctions. Electrical measurement results show that the as-grown graphene/Si heterojunction has a typical current rectification behavior, indicating a Schottky barrier between graphene and Si. Moreover, the graphene/Si based near-infrared photodetector with outstanding photovoltaic conversion capability exhibits high-performances in responsivity (50 mA W^−1^) and detectivity (∼1.1 × 10^10^ cm Hz^1/2^ W^−1^) when illuminated with a 1550 nm light.

## 2. Materials and methods

**Fig. 1:**
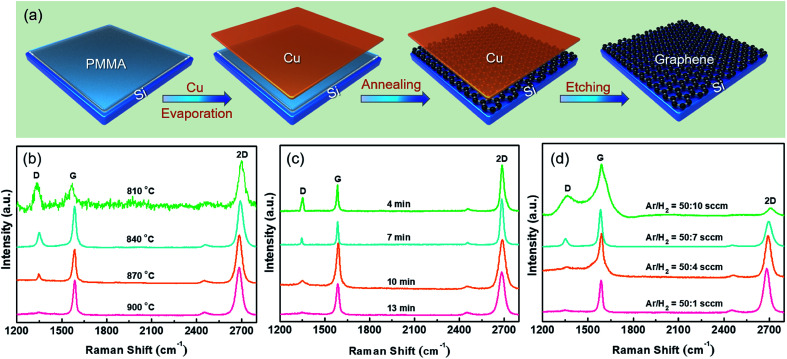
Condition optimization for graphene preparation. (a) Schematic process flow of synthesizing graphene on Si. (b–d) Raman results of the as-grown graphene on Si at various grown conditions: (b) temperatures, (c) annealing times, and (d) Ar/H_2_ ratios.

Fig. 1a displays the schematic process flow for directly synthesizing graphene on Si. After cleaning the Si substrate by Radio Corporation of American (RCA), the native oxide layer was removed by buffered oxide etch (BOE). A 200 μL PMMA (MicroChem Corp. 950, A4) in toluene with a concentration of 0.025 wt% yielded about 2 nm-thick PMMA film at a spin-coat rate of 8000 for 100 s. The thicknesses of PMMA film was determined by ellipsometry. Then a 30 nm-thick Cu layer was deposited on the Si/PMMA as the catalyst. Subsequently, the Si/PMMA/Cu sample was loaded into a CVD chamber for thermal annealing under a H_2_/Ar flow, and graphene was formed and sandwiched by Si substrate and Cu capping layer. After the removal of Cu layer by chemical etching process in 20% (NH_4_)_2_S_2_O_8_ aqueous solution, graphene is exposed on the Si substrate.

The growth condition for high-quality graphene is optimized by tuning the annealing temperature, growth time, and gas atmosphere. For materials characterizations, the layer number, quality, and uniformity of the as-grown graphene were evaluated by Raman scattering (HORIBA Jobin Yvon HR800), atomic force microscopy (AFM, Multimode 8) measurement and transmission electron microscope (TEM, FET-Tecnai G2F20 S-7WIN); the element distribution along the depth of the as-grown graphene was analyzed by secondary ion mass spectrometry (SIMS, Cameca IMS-4F, Paris, France); the surface chemical composition of as-grown graphene was revealed by X-ray photoelectron spectroscopy (XPS, PHI 5802, Physical Electronics Inc, Eden Prairie, MN); the crystalline quality and microstructure of the as-grown graphene were accessed by scanning tunneling microscope (STM, SPECS JT-STM) in the constant current mode; electrical properties of graphene/Si based devices were investigated *via* a semiconductor parameter analyzer (B1500A and Keithley 4200).

## 3. Results and discussion

### 3.1 Condition optimization for graphene preparation

Fig. 1b indicates the Raman results of the graphene grown at different temperatures of 810, 840, 870 and 900 °C. At 810 °C or lower, three representative peaks locating at 1350 cm^−1^, 1580 cm^−1^, and 2700 cm^−1^ are found, which are known as the characteristic peaks of graphene,^8,9^*i.e.*, D-peak, G-peak and 2D-peak, respectively. The calculated peak intensity ratio, *I*_D_/*I*_G_, of graphene synthesized at a temperature of 810 °C is about 1, implying the presence of defects.^18^ As the growth temperature increases, the intensity of D-peak gradually attenuates, suggesting an improved crystalline quality. Notably, no appreciable D-peak is observed at 900 °C. Therefore, the optimized temperature for graphene growth is 900 °C. In addition to the annealing temperature, the annealing time is also regarded as an important factor for obtaining graphene with high-quality.^19^ With an annealing time of 4 min, a distinct D-band is observed, as shown in Fig. 1c. As the annealing time increasing, the D-peak is suppressed to disappear when annealed for 13 min. Prolonging the annealing time, such as 15 min or longer, the obtained Raman spectra (not shown here) are similar to the sample annealed for 13 min. Previous works demonstrated that PMMA starts to decompose even the annealing temperature is low (∼600 °C),^14^ therefore, complete PMMA decomposition and graphene formation should need sufficient annealing temperature and time. Once the annealing time is over 13 min, the graphene quality will not be further improved. The growth condition was further optimized by changing the Ar : H_2_ flow rate ratio (sccm), as shown in Fig. 1d. The annealing temperature and time are fixed at 900 °C and 13 min. As the Ar : H_2_ flow rate ratio is increased from 50 : 10 to 50 : 1, the intensity of D-peak for graphene decreases rapidly, indicating an improved crystalline quality. When the H_2_ flow is reduced to 1 sccm, the D-band completely disappears.

### 3.2 The preparation of graphene on Si and its characterization

**Fig. 2:**
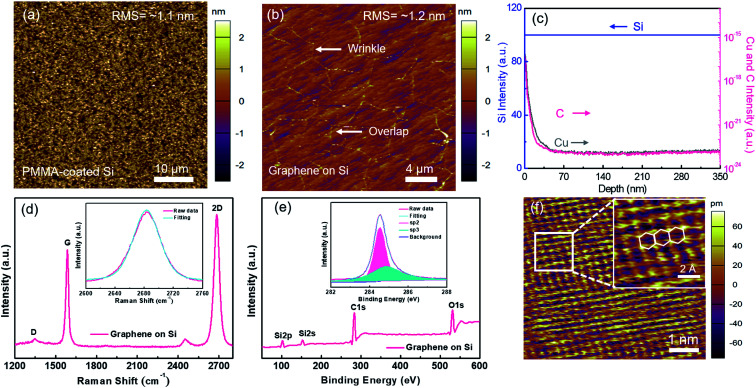
The preparation of graphene on Si and its characterization. Surface morphology and roughness analyses of: (a) PMMA-coated Si substrate and (b) as-grown graphene on Si substrate. (c) SIMS depth profiles of Cu atoms in Si substrate. Cu/PMMA/Si system after graphene growth and Cu layer removal. (d) Raman spectrum of as-grown graphene on Si substrate under the optimized condition; Lorentzian fitting of the 2D band is performed to determine the FWHM (inset). (e) XPS analysis of graphene/Si after removing Cu. (f) STM image of as-grown graphene on Si.

In our experiment, water-solvable PMMA was selected because of its capability of forming large-area uniform film with tunable thickness on various substrates by spin-coating.^20^ A conformal PMMA coating layer on the desired substrate has been demonstrated as a vital step for synthesizing high-quality graphene, which can be attributed to the intimate relationship between the layer number of graphene and the carbon distribution in PMMA.^21,22^ In order to verify the uniformity of PMMA spin-coated on Si, AFM measurement was performed. As shown in Fig. 2a, the surface roughness of PMMA is about 1.1 nm over a scanned area of 45 × 45 μm^2^. The surface roughness has no significant change after exposing graphene which is formed under the above optimized condition (temperature: 900 °C; time: 13 min; H_2_ to Ar flow rate ratio: 50 : 1), as demonstrated in Fig. 2b with a value of about 1.2 nm. The residual Cu atoms in graphene/Si were investigated by secondary ion mass spectrometry (SIMS). As displayed in Fig. 2c, the fraction of Cu atoms residual in the Si substrate is extremely low with a concentration even below the detection limit of the instruments. We also notice that carbon atoms are also found in the Si substrate although the fraction is rather low.

Fig. 2d displays the Raman spectrum of the as-grown graphene synthesized at the optimized condition. The strongly suppressed D-peak indicates that the as-grown graphene has a comparable quality to the graphene grown by conventional CVD.^9^ From the Lorentzian fitting curve of 2D-peak (see inset of Fig. 2d), the corresponding full-width at half-maximum (FWHM) is extracted as about 35 cm^−1^, evidencing the optimized graphene is monolayer.^18^ From the XPS analysis of the as-grown sample shown in Fig. 2e, Si, carbon (C) and oxygen (O) characteristic peaks are found. A sp^2^-hybridization peak of carbon, locating at ∼284.3 eV, is exposed from the deconvolution of C-1s spectrum (inset, Fig. 2e). Therefore, one can conclude that the sp^2^ network, *i.e.*, graphene, is formed.^23^ The minor peak locating at ∼285.3 eV denotes the sp^3^-hybridization is generated from the natural carbon contamination.^24^Fig. 2f shows the STM measurement of the as-grown graphene, and the magnified high-resolution STM result, with a representative honeycomb lattice structure,^18^ is displayed in the inset. No visible lattice defect is observed within the area greater than 8 nm × 8 nm, indicating the high crystalline quality of the as-synthesized graphene.

### 3.3 Thickness and electrical property evaluation of the synthesized graphene

**Fig. 3:**
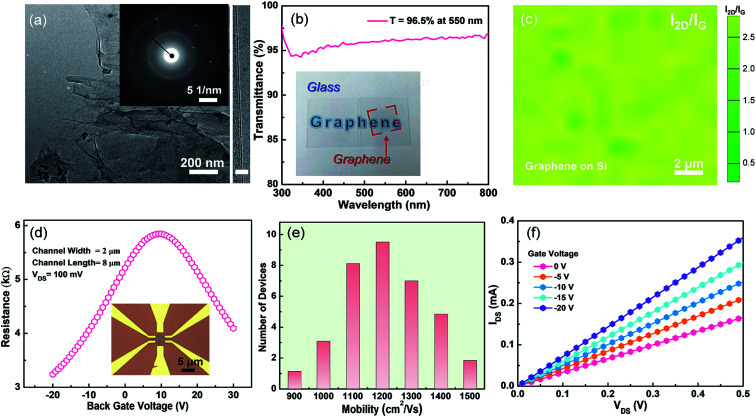
Thickness and electrical property evaluation of the as-synthesized graphene. (a) TEM (left; inset: SAED pattern) and HR-TEM (right) images of the as-grown graphene on Si substrate. Scale bar is 3 nm. (b) Optical transmittance of the directly grown monolayer graphene on glass substrate. A digital image of graphene on 1 × 1 cm^2^ quartz is displayed in the inset. (c) Raman mapping results of *I*_2D_/*I*_G_ measured from the as-grown graphene. (d) *I*_DS_–*V*_G_ curves of back-gated GFET with directly grown monolayer graphene on SiO_2_/Si substrate as channel, *V*_DS_ = 100 mV. A typical device image is shown in the inset. (e) Histogram of the mobility distribution acquired from 36 GFETs. (f) Output (*I*_DS_–*V*_DS_) characteristics of the GFET at different *V*_G_.

Fig. 3a shows the TEM (left panel) image of the synthesized graphene, and a continuous graphene over a large area is observed. From the selected area electron diffraction (SAED, left inset) result that one set of hexagonal diffraction pattern is presented, the as-grown graphene has a single-crystalline lattice structure. The layer number of the as-grown can be intuitively confirmed by the high-resolution TEM (HR-TEM) result, as shown in the right of Fig. 3a. Only one straight line is observed, indicating the as-grown graphene is monolayer. In our work, direct synthesis of monolayer graphene on arbitrary substrate (such as SiO_2_ and glass) by utilizing copper-assisted chemical vapor deposition, without any post-transfer process for optical transmittance and transport properties measurements. To further investigate the layer number, the optical transmittance of as-grown graphene on glass is characterized and the result is presented in Fig. 3b. The transmittance is 96.5% at the wavelength of 550 nm. Because the absorbance for an individual graphene layer is about 2.3%,^25^ the layer number of as-grown graphene can be estimated to be only one atom layer, which is consistent with HR-TEM and Raman results. Moreover, the inset of Fig. 3b shows that monolayer graphene has good transparency and macroscopic uniformity. To determine the quality and uniformity, a random area 30 μm × 30 μm of the as-grown graphene is selected for Raman mapping measurement. Fig. 3c shows the extracted ratios of 2D to G peak intensity. The *I*_2D_/*I*_G_ is in a range of 2–2.5, conforming the graphene grown on Si substrate by the presented method is homogenous and uniform.

Good transport properties, especially the carrier mobility, of graphene determines its potentials for the applications in high-performance electronic devices. In order to investigate the electrical transport ability of the graphene grown by our proposed method, graphene-based field effect transistors (GFETs) with a back gate were fabricated and characterized. Fig. 3d shows a typical resistance-gate voltage curve measured from the GFET. After measuring 36 GFETs, the calculated hole mobility mainly locates at 1000–1200 cm^2^ V^−1^ s^−1^, and the electron mobility is in the range of 1200–1400 cm^2^ V^−1^ s^−1^, are shown in Fig. 3e. We should stress that these carrier mobility values for hole and electron are comparable to recently reported values measured from the CVD graphene.^9,18,26^Fig. 3f shows the output characteristics of GFETs. Under arbitrary biased gate voltage, *I*_DS_ always increases linearly with *V*_DS_. Therefore, the contact type between electrode (Ti/Au) and graphene is ohmic. Notably, *I*_DS_ decreases monotonously with the increase of *V*_G_ swapping from −20 to 0 V, implying a slight p-doping in the as-synthesized graphene.^27^

### 3.4 Responsivity and detectivity of graphene/Si based photodetector

**Fig. 4:**
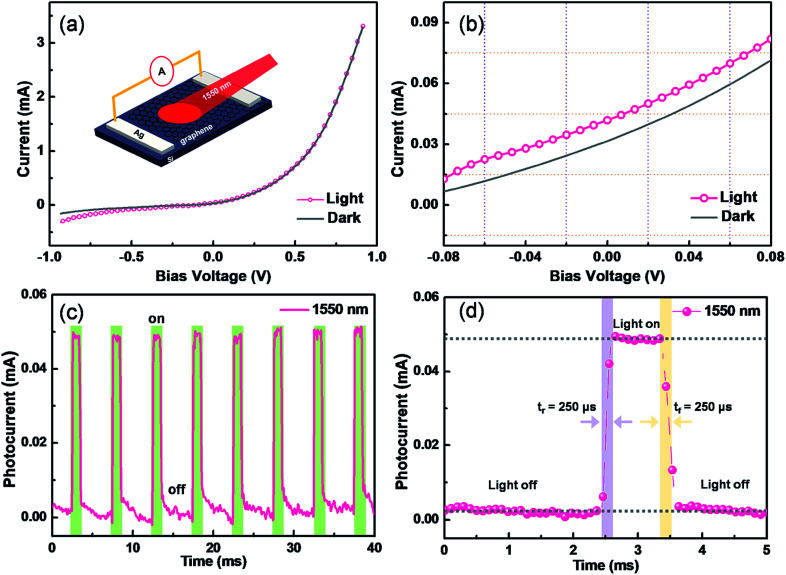
Responsivity and detectivity of graphene/Si based photodetector. (a) Currents of the graphene/Si based photodetector as a function of bias voltage with/without light illumination. A schematic illustration of the photodetector is shown in the inset. (b) The magnified *I*–*V* characteristic in a low voltage range. (c) Photocurrent of the photodetector illuminated with a pulsed 1550 nm light, and the power density is about 27 mW cm^−2^. (d) Enlarged photovoltage of the graphene/Si Schottky photodetector responded to a pulsed illumination.

Photodetectors have exhibited enormous potentials in optical communications, biomedical imaging, and motion detection.^28^ In terms of high-performance optoelectronic devices, graphene has attracted significant attentions due to its superior electronic and optical properties. For our proposed method, the significant advantage is directly integrating the as-grown graphene/Si heterojunction into a Schottky junction-based photodetector, because the average Schottky barriers of graphene/silicon junctions are estimated to be 0.45–0.47 eV.^10^ The performances of graphene/Si photodetectors are assessed by measuring the current passing through the Schottky heterojunction at dark and light illumination conditions, as shown in Fig. 4a. Without the illumination, typical rectifying behavior is observed, further confirming the contact between graphene and Si is Schottky-type.^29,30^ When illuminated by a 1550 nm light, the reversed current is significantly enhanced.^31^ To investigate more details, a magnification of the *I*–*V* characteristic is processed and shown in Fig. 4b, and a typical photovoltaic effect is found.^10^Fig. 4c depicts the photocurrent of a graphene/Si photodetector varying with the illumination from a pulsed 1550 light. The bias voltage is set as zero. Highly reversible low- or high-resistivity states are demonstrated. Fig. 4d shows the enlarged transient photo-response curve, where both rise time (*t*_r_) and fall time (*t*_f_) are estimated to be only 250 μs. Moreover, the detectivity and responsivity are calculated to be as high as ∼1.1 × 10^10^ cm Hz^1/2^ W^−1^ and ∼50 mA W^−1^, respectively, suggesting great potentials in logic and photoelectric circuits.

## 4. Conclusions

In summary, we describe a simple and convenient approach to directly synthesize graphene on single-crystalline silicon substrate. The quality of synthesized graphene is optimized *via* tuning the growth conditions. To investigate the optoelectronic properties, Schottky junction based photodetectors are directly constructed by utilizing the as-grown graphene/Si. Optoelectronic results indicate that the as-grown graphene/Si based photodetector exhibits a distinct photovoltaic behavior, with high responsivity and detectivity of ∼50 mA W^−1^ and ∼1.1 × 10^10^ cm Hz^1/2^ W^−1^, respectively. Our study may pave the way for synthesizing graphene on arbitrary substrate in a highly scalable way, thus promoting applications of graphene in microelectronic and optoelectronic fields.

## Conflicts of interest

The authors declare that they have no conflict of interest.

## Supplementary Material
